# Pediatric Open Long-Bone Fracture and Subsequent Deep Infection Risk: The Importance of Early Hospital Care

**DOI:** 10.3390/children9081243

**Published:** 2022-08-17

**Authors:** Andrew W. Kuhn, Stockton C. Troyer, Jeffrey E. Martus

**Affiliations:** 1 Department of Orthopedic Surgery, Washington University in St. Louis, St. Louis, MO 63108, USA; 2 Washington University School of Medicine in St. Louis, St. Louis, MO 63108, USA; 3 Department of Orthopaedic Surgery and Rehabilitation, Vanderbilt University Medical Center, Nashville, TN 37232, USA

**Keywords:** pediatric, open fracture, infection, trauma, morbidity, orthopedic

## Abstract

The purpose of the current study was to identify risk factors for deep infection after an open long-bone fracture in pediatric patients. Systematic billing queries were utilized to identify pediatric patients who presented to a level I trauma center from 1998 to 2019 with open long-bone fractures. There were 303 open long-bone fractures, and 24 (7.9%) of these became infected. Fractures of the tibia/fibula (*p =* 0.022), higher revised Gustilo-Anderson type (*p =* 0.017), and a longer duration of time between the injury and hospital presentation (*p =* 0.008) were all associated with the presence of deep infection. Those who went on to have a deep infection also required more operative debridements (*p =* 0.022) and a total number of operative procedures (*p =* 0.026). The only factor that remained significant in multivariable regression was the duration between the injury and hospital presentation (OR 1.01 [95%CI 1.003–1.017]; *p =* 0.009), where the odds of deep infection increased by 1% for every minute of delayed presentation.

## 1. Introduction

Fractures represent a significant proportion of all pediatric emergency department visits and hospital admissions in the United States, especially for older male adolescents [1,2]. In a study of 3350 children with 3413 limb fractures presenting to one center, distal radius fractures, supra-condylar fractures of the humerus, and forearm shaft fractures were most common, while femur and tibia/fibula fractures also accounted for a large proportion of fractures in other studies [2,3]. Although open fractures are thought to contribute a small percentage (<10%) of all pediatric fractures, they are considered surgical emergencies as they carry a significant risk for infection and associated morbidity [4,5,6].

Open fractures are typically stratified by the revised Gustilo–Anderson Classification, where the type (I, II, and IIIA-C) is based on wound size and the extent of associated tissue damage [7]. A treatment protocol for open fractures first described and employed by Gustilo and Anderson between 1969 and 1973, portended a significant reduction in infection rates through debridement and copious irrigation, primary closure for type I and II fractures and secondary closure for type III fractures, no primary internal fixation except in the presence of associated vascular injuries, cultures of all wounds, and oxacillin-ampicillin before and for three days postoperatively [7,8]. Some aspects of pediatric open fracture management remain unchanged and are universally accepted across institutions, such as immediate antibiotic administration and tetanus prophylaxis, whereas other facets of care are more controversial and debated, particularly in the setting and management of type I open fractures [9,10,11,12].

In an attempt to optimize hospital course and reduce the risk of complications and infection following pediatric open fractures, different aspects of management have been empirically studied including: the initiation and duration of antibiotic treatment [13,14]; choice of antibiotics and the utility of preoperative cultures [13,14,15,16]; surgical approach for all fractures and nonoperative treatment for type I fractures [12,13,14,17,18,19,20,21,22,23,24,25,26]; time to operative debridement and irrigation [13,14,27,28,29]; the addition of negative pressure dressings [13,30,31]. Unfortunately, when compared to the management of open fractures in adults, high-level evidence is lacking, and most recommendations are based on case-series and/or historical standards of care [6,13,14]. For instance, the most recent published review and recommendations for antibiotic selection in pediatric open fractures found a paucity of high-level evidence and concluded broadly that Type I open fractures should be treated with a first-generation cephalosporin and for type II and III, additional Gram-negative coverage should be added [32].

The primary aim of this study is to describe trends in management and elucidate pertinent risk factors for developing a deep infection after an open long-bone fracture in pediatric patients. We hypothesized that infection would be more prevalent in higher revised Gustilo–Anderson type open fractures as well as in cases of delayed administration of antibiotic prophylaxis.

## 2. Materials and Methods

### 2.1. Patient Identification, Inclusion and Exclusion Criteria

Patients under the age of 18 who presented to Vanderbilt University Medical Center (VUMC) between 1998 and 2019 with an open fracture of a long bone were retrospectively queried with over 1000 ICD9 and ICD10 codes pertaining to open long-bone fractures. Patients were only included if they had a documented open long bone fracture event and adequate data and follow-up. Traumatic amputations were not included in this analysis. If patients did not have documentation of their “date and time of injury”, “date and time of admission to an outside facility (OSF)/VUMC”, or “date, time, and duration of antibiotics administered”, they were excluded. Patients had to have follow-up through deep infection, “healed fracture” (near or complete union), or fracture non-/malunion to be included in this study. If patients were discharged from a surgeon’s care and instructed to follow up on an “as needed” basis, they met the necessary endpoint and were considered healed. If a patient had multiple open long bone fractures resulting from the same injury, each was treated as a unique event. Our institutional antibiotic protocol is as follows: Type I or II pediatric open fractures are treated with cefazolin. If allergic to cefazolin, clindamycin is given instead. If allergic to both cefazolin and clindamycin, vancomycin is initiated. For type III or highly contaminated type I or II pediatric open fractures, piperacillin/tazobactam is administered. If allergic to piperacillin/tazobactam, clindamycin or vancomycin is provided, with metronidazole and either ciprofloxacin or gentamicin. Antibiotics are administered within 1 h of arrival. A tetanus vaccine is also offered if not up to date.

### 2.2. Database Structure and Elements

Demographic and injury characteristics, management decisions, the temporality of various treatments, and outcome variables were collected and based on previously developed data collection instruments (Registry for Orthopaedic Trauma in Children, ROTC). Additional elements that were thought to be possibly associated with infection were included as well. All study data were collected, managed, and built using the REDCap electronic data capture tool hosted at VUMC [33]. REDCap is a secure, web-based application designed to support data capture for research studies, providing: (1) an intuitive interface for validated data entry; (2) audit trails for tracking data manipulation and export procedures; (3) automated export procedures for seamless data downloads to common statistical packages; (4) procedures for importing data from external sources.

For date–time event variables, scanned Emergency Medical Services (EMS), triage notes, other documents and clinical notes, and documentation of different services (e.g., anesthesia care records) and/or procedure notes were utilized to ensure the most accurate date–times possible. For “date and time of discharge”, 12:00 p.m. was utilized given the lack of precise timing documented. Deep infections were based on clinical notation and/or direct confirmatory signs (fistula, sinus, wound breakdown, purulent drainage or pus, positive cultures, or histopathological examination). All risk factors were in relation to each patient’s endpoint. Each variable was assessed in its relation to developing a deep infection, and how they were defined for the purposes of this study can be found in Table 1. This is a historical cohort in which data from medical records were reviewed and collected. Institutional Review Board (IRB) approval was granted (VUMC; #182036) for this study.

### 2.3. Statistical Analyses

Pertinent demographics, injury characteristics, management decisions, the temporality of various treatments, and outcomes across cases were described and reported utilizing raw counts, measures of central tendency (mean, median, or mode), and measures of data dispersion (95% confidence intervals, standard errors, inter-quartile ranges) where appropriate. First, univariate logistic regression modeling was conducted to assess the associations between all relevant independent variables and the presence of a deep infection. Associations that reached a threshold of *p* < 0.05 were entered into a multivariable logistic regression model. To account for the rarity of deep infection and the phenomenon of separation, Firth’s correction was utilized to adjust for biased estimates by maximizing the penalized likelihood function [34]. Effect sizes were reported as odds ratios (OR) with 95% confidence intervals. All statistics were computed with SPSS v26.0 (IBM; Armonk, NY, USA).

## 3. Results

### 3.1. Patient Characteristics

**Figure 1:**
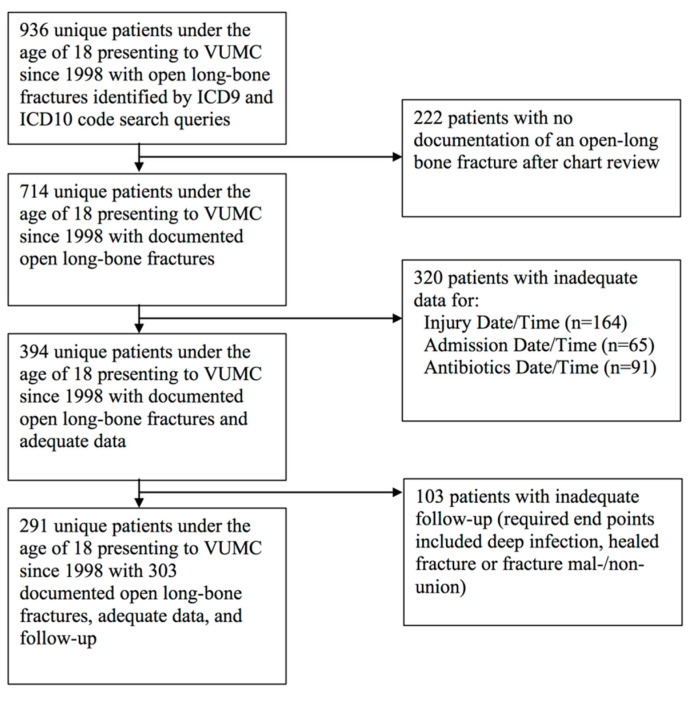
Inclusion and Exclusion Flowchart.

Nine hundred and thirty-six patients were returned from the systematic billing queries, and 291 met the necessary inclusion and exclusion criteria (Figure 1). There were 303 open long-bone fractures, and 24 (7.9%) of these became infected. Patients were, on average, 11.8 (±4.2) years old at the time of presentation. Most were male (65.0%), white (73.3%), and had no documented systemic, metabolic, skeletal, or psychiatric/neurologic comorbidities (73.3%). Around 15% had a self-reported antibiotic allergy. Patients who had a deep infection were typically older (12.4 vs. 11.8 years old), more likely to be male (79.1% vs. 63.8%), and have comorbidities at presentation (37.5% vs. 25.8%). However, none of these or the other collected patient demographic characteristics were significantly associated with deep infection (Table 2).

### 3.2. Open Fracture and Injury Event Characteristics

Most of the fractures occurred in the summer (38.0%) and spring (27.1%), resulting from high-energy mechanisms (56.4%) in high-risk settings (49.1%). A large proportion (40.6%) of patients presented with significant head, chest, abdominal, or other injuries requiring additional work-up and treatment. The most commonly fractured long bone was the radius/ulna (42.9%), followed by the tibia/fibula (32.3%). Most fractures were diaphyseal (71.0%) and type I or II open fractures (70.6%). Vascular compromise/injury (8.6%), nerve injury (12.5%), and compartment syndrome requiring fasciotomy (5.9%) were less frequent. Long-bone fracture was significantly associated with the occurrence of deep infection (*p =* 0.022). The tibia/fibula was significantly more likely to become infected compared to the radius/ulna (OR = 4.00 [1.515–12.05]). Revised Gustilo–Anderson classification was also significantly associated with deep infection (*p =* 0.017), whereas compared to type I, type IIIA-C fractures were at over four times the odds of developing deep infection (OR = 4.411 [1.559, 14.984]) (Table 3).

### 3.3. Open Fracture Management Characteristics

Most patients presented to an OSF or VUMC a little over an h (64.0 (±44.0) min) from their injury. Close to a third (32.0%) were transferred to VUMC from an OSF for definitive care. Most patients (73.9%) received antibiotics within 3 h of their injury (161.4 (±152.8) min) and received a median of 1 (IQR: 1-2) antibiotic class during their admission. The mean time to operative debridement was a little under 15 h from their injury (14.8 (±11.1) h). Approximately one-fifth (17.9%) of open fractures were operatively debrided within 6 h of injury, and the median number of operative debridements was 1 (IQR 1-1). The time to definitive fixation was a little over a day (24.5 (±36.9) h). The average duration between first and last antibiotic administration while admitted was almost 3 days (69.8 (±102.7) h), and the average length of hospital stays was about 4 days (4.0 (±4.8) days). The majority of patients (94.7%) were discharged home, and around one-fifth (19.9%) were on antibiotics. The total number of operative procedures needed before each patient’s endpoint was 1 (IQR 1-1). Those who had longer durations of time between their injury and presentation to either an OSF or VUMC were more likely to become infected (89.9 vs. 61.8 min; OR 1.009 [95%CI 1.003, 1.016]; *p =* 0.008). The duration of time between injury and presentation to either an OSF or VUMC was significantly correlated with the duration between the injury and first antibiotic administration (r = 0.176, *p =* 0.002), but not with the duration of time between the injury and first operative irrigation and debridement (r = −0.036, *p =* 0.538). Those who went on to have deep infections required more operative debridements (*p =* 0.022) and a greater number of operative procedures (*p =* 0.026) (Table 4).

### 3.4. Multivariable Analysis

**Figure 2:**
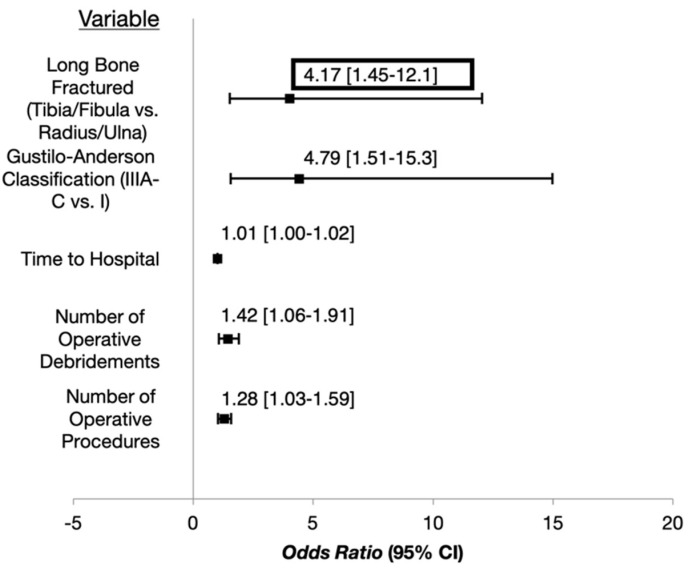
Odds Ratios and 95% Confidence Intervals for Factors Significantly Associated with D-veloping a Deep Infection After Pediatric Open Long-Bone Fractures.

There were five factors significantly associated with the development of deep infection after an open long bone fracture (Figure 2). When these factors were incorporated into a single multivariable regression model, the only factor that remained significant was the duration of time between the injury event and presentation to an OSF or VUMC, where for every minute that time to hospital presentation was delayed, the odds of deep infection increased by 1% [OR = 1.010 (1.003–1.018); *p =* 0.006] (Table 5).

## 4. Discussion

The purpose of the current study was to identify factors associated with developing a deep infection in pediatric patients with open long-bone fractures. Five variables were independently associated: (1) Fractures of the tibia/fibula; (2) higher revised Gustilo–Anderson type; (3) a longer duration of time between the injury and presentation to a hospital; (4) a higher number of operative debridements; (5) a higher number of total operative procedures. After incorporating all significant variables into a multivariable regression model, the only variable that remained statistically significant was the duration of time between the injury and presentation to a hospital, where for every additional minute delay in hospital presentation, the odds of infection increased by 1%.

Unfortunately, the literature regarding infection risk after pediatric open fracture is sparse and comprised primarily of small case series or small cohorts addressing a single risk factor [13]. In a recent systematic review of the adult literature, lower extremity open fractures were significantly more likely to develop infectious complications compared to upper extremity fractures [35]. A recent systematic review and meta-analysis of pediatric open fractures found that Gustilo–Anderson type III fractures of the tibia were associated with a lower risk of osteomyelitis than femoral fractures and found the lowest rates of osteomyelitis/infection in upper limb fractures [12]. Our study similarly found that the lower extremity, specifically the tibia/fibula is at greater risk for deep infection after an open fracture in the pediatric population. Additionally, our study demonstrated that the infection risk for type IIIA-C open fractures is four times greater compared to type I open fractures, a trend that was previously demonstrated in a 2009 systematic review by Baldwin et al. [36]. Their pooled analyses revealed that type III open tibia fractures in children were 3.48 times more likely to have an infectious complication compared to type I fractures and 2.28-fold more likely compared to type II. Interestingly, when Luhmann et al. [37] analyzed 65 open forearm fractures in a pediatric sample, fracture type was not associated with infection; however, as the authors note, the study may not have had adequate power to detect statistically significant differences.

Immediate antibiotic administration has been heralded as a mainstay of open fracture management. Patzakis and Wilson [38] demonstrated an infection rate of 4.7% for open fractures when antibiotics were administered within 3 h after injury and a rate of 7.2% when there was a delay of ≥3 h. However, they included both pediatric and adult patients. In the current study of only pediatric patients, the average time to antibiotics was under 3 h [161.5 (±157.4) min] for all patients, and there was no relationship between time to antibiotics and the development of a deep infection when the variable was left as a continuous measure or when patients were binned into two groups, <3 h vs. ≥3 h. However, time to first antibiotic administration was significantly associated (although with a small effect size) with time to hospital presentation in our post hoc correlative analyses, thus supporting the importance of early antibiotic administration. It has also been the classic teaching to debride the wound within 6 h of injury. Time to operative debridement was, on average, 14.5 (±8.5) h. There was no relationship between time to operative debridement and the presence of deep infection when the variable was left as a continuous measure or when patients were binned into two groups, <6 h vs. ≥6 h. These results are similar to two previously published studies by Skaggs et al. [28,29], who looked at 554 and 118 pediatric open fractures, finding that infection rates were not related to time to operative debridement groups of <6 h, 7–24 h, 25+ h and <6, 6–12, 12–24, and 24+ h, respectively. Kelly et al. also found no association between the development of infection and time to surgical debridement in 288 open fractures in pediatric patients [6]. Ibrahim et al. [27], 2014, examined the effect of delayed surgical debridement in pediatric open fractures by conducting a systematic review and meta-analysis. Late surgical debridement was associated with a pooled rate of infection of 2.5%, which was not higher than the infection rate of 4.2% seen for early surgical debridement (<6 h) in children with open fractures. The number of total operative debridements and operative procedures was associated with the presence of a deep infection in our study, which may represent a more correlative than causative relationship.

Even though time to antibiotics and time to operative debridement were not independently associated with the presence of deep infection in this study, the duration of time between the injury event and presentation to a hospital was a risk factor. For every additional minute delay in hospital presentation, the odds of infection increased by 1%. This finding raises more questions than answers and requires further study, given that the data included in this retrospective study were limited in both granularity and power. Factors surrounding immediate open fracture management in the field and in the emergency department should be included in future studies, including the performance and timing of temporary wound coverage with a sterile bandage, bedside irrigation and/or debridement, closed reduction, splinting, etc.

There are other limitations in this study worth declaring. As previously mentioned, a low number of cases required that data be binned and categorized into a smaller number of groups in order to maintain adequate power. Therefore, we could not analyze trends that required greater detail, such as antibiotic type and dosing. This resulted in analyzing antibiotic therapy as a count variable based on the number of different antibiotic classes administered. In a similar manner, we could not analyze factors related to definitive fixation and soft tissue coverage with substantial detail. Our search strategy may also not have identified every patient with an open fracture over the last two decades due to inaccurate billing codes. Lastly, retrospective research relies on reviewing records, which may have contained both factual and temporal inaccuracies. Future prospective and collaborative research should aim to identify additional risk factors for deep infection and clarify the ideal treatment strategy for these injuries. This will advance efforts to minimize complications and optimize outcomes for children who sustain open long-bone fractures.

## 5. Conclusions

Deep infection was associated with open fractures of the tibia/fibula and higher type open fractures. For every additional minute delay in hospital presentation, the odds of infection increased by 1%, suggesting that early hospital care is a critical factor in the management of these injuries. We recommend that in all cases of potential open fracture, children should present to a hospital as quickly as possible to be evaluated, receive care, and reduce the risk of deep infection.

## Figures and Tables

**Table 1:** Variables Assessed in Relation to Acquiring a Deep Infection After Pediatric Open Long-Bone Fracture.

Characteristic	Coded
Age	(Continuous Measurement)
Sex	Female, Male
Race ^a^	White, Non-White
Weight ^b^	(Continuous)
Comorbidities ^c^	No, Yes
Self-Reported Antibiotic Allergies	No, Yes
Season ^d^	Spring, Summer, Fall, Winter
Mechanism of Injury ^e^	Low Energy, High Energy
Setting and Contamination Risk ^f^	Low Risk, Medium Risk, High Risk
Polytrauma ^g^	No, Yes
Multiple Open Fractures	No, Yes
Long-Bone Fractured	Femur, Humerus, Radius/Ulna, Tibia/Fibula
Segment of Long Bone Fractured	Diaphyseal, Metaphyseal/Epiphyseal
Revised Gustilo-Anderson Classification ^h^	I, II, IIIA-C
Vascular Compromise/Injury	No, Yes
Nerve Injury	No, Yes
Compartment Syndrome Requiring Fasciotomy	No, Yes
Time to Admission (min)	(Continuous Measurement)
Transferred From Outside Facility	No, Yes
Time to Antibiotics (min)	(Continuous Measurement), < or ≥3 h
Number of Antibiotic Classes Administered	(Count Measurement)
Time to Operative Debridement (h)	(Continuous Measurement), < or ≥6 h
Number of Operative Debridements	(Count Variable)
Time to Definitive Fixation (h)	(Continuous Measurement)
Duration of Antibiotics While Admitted (h)	(Continuous Measurement)
Length of Hospital Stay (days)	(Continuous Measurement)
Discharged with Antibiotics	No, Yes
Discharge Disposition	Home, Rehabilitation/Other
Total Number of Operative Procedures	(Count Measurement)

^a^ White or Non-White (Black or African American, Asian, Native Hawaiian or Other Pacific Islander, American Indian or Alaska Native, of Spanish Origin, or Other). ^b^ Standardized to z-scores based on sex and age-adjusted normative data https://web.emmes.com/study/ped/resources/htwtcalc.htm (accessed on 1 October 2019). ^c^ Harboring one or more documented systemic, metabolic, skeletal, or psychiatric/neurologic conditions on presentation. ^d^ Spring (March, April, May), Summer (June, July, August), Fall (September, October, November), Winter (December, January, February). ^e^ High Energy (MVC, ATV, MCC, other machinery-related, crush-related, GSW, fall > 10 feet), Low Energy (fall < 10 feet, sport-related, bicycle, monkey bars, trampoline “rough-housing”). ^f^ High Risk (outside barnyard, fecal, dirty water, dirt/grass, mulch, playground, or a deeply contaminated wound); Medium Risk (outside street, pavement, concrete, hard surface, or surface contamination of the wound); Low Risk (inside with minimal or no wound contamination). ^g^ Presenting with significant head, chest, abdominal, or other injuries requiring additional work-up and treatment. ^h^ Designated by the attending orthopaedic surgeon in nearly all cases and based on clinical history, presentation, exam, imaging, and intraoperative findings.

**Table 2:** Patient Demographic Characteristics.

Characteristic Mean (SD), Median (IQR), or *n* (%)	No Infection (*n* = 279)	Deep Infection (*n* = 24)	TOTAL (*n* = 303)	OR [95%OR]	*p*-Value
Age	11.8 (±4.2)	12.4 (±4.7)	11.8 (±4.2)	1.029 [0.934, 1.143]	0.563
Sex					
Female *	101 (36.2)	5 (20.8)	106 (35.0)		
Male	178 (63.8)	19 (79.1)	197 (65.0)	2.015 [0.807, 5.912]	0.139
Race					
White *	205 (73.5)	17 (70.8)	222 (73.3)		
Non-White	74 (26.5)	7 (29.2)	81 (26.7)	1.182 [0.456, 2.801]	0.717
Weight (Standardized Z-Score)	0.4 (±1.2)	0.6 (±1.3)	0.4 (±1.2)	1.182 [0.834, 1.687]	0.349
Comorbidities					
No *	207 (74.2)	15 (62.5)	222 (73.3)		
Yes	72 (25.8)	9 (37.5)	81 (26.7)	1.754 [0.725, 4.051]	0.205
Self-Reported Antibiotic Allergies					
No *					
Yes	237 (84.9) 42 (15.1)	19 (79.2) 5 (20.8)	256 (84.5) 47 (15.5)	1.576 [0.527, 4.051	0.390

* reference group.

**Table 3:** Open Fracture and Injury Event Characteristics.

Characteristic Mean (SD), Median (IQR), or *n* (%)	No Infection (*n* = 279)	Deep Infection (*n* = 24)	TOTAL (*n* = 303)	OR [95%OR]	*p*-Value
Season					0.279
Winter *	34 (12.2)	1 (4.2)	35 (11.6)		
Spring	75 (26.9)	7 (29.2)	82 (27.1)	2.284 [0.472, 22.26]	331
Summer	102 (36.6)	13 (54.2)	115 (38.0)	3.028 [0.698, 28.42]	0.154
Fall	68 (24.4)	3 (12.5)	71 (23.4)	1.175 [0.185, 12.47]	0.870
Mechanism of Injury					
Low Energy *	126 (45.2)	6 (25.0)	132 (43.6)		
High Energy	153 (54.8)	18 (75.0)	171 (56.4)	2.347 [0.974, 6.379]	0.057
Setting and Contamination					0.239
Low Risk *	58 (22.4)	3 (12.5)	61 (21.6)		
Medium Risk	78 (30.1)	5 (20.8)	83 (29.3)	1.171 [0.299, 5.228]	0.822
High Risk	123 (47.5)	16 (66.7)	139 (49.1)	2.232 [0.750, 8.785]	0.158
Polytrauma					
No *	168 (60.2)	12 (50.0)	180 (59.4)		
Yes	111 (39.8)	12 (50.0)	123 (40.6)	1.511 [0.660, 3.463]	0.325
Multiple Open Fractures					
No *	256 (91.8)	23 (95.8)	279 (92.1)		
Yes	23 (8.2)	1 (4.2)	24 (7.9)	0.697 [0.075, 2.900]	0.663
Long-Bone					0.022 **
Tibia/Fibula *	84 (30.1)	14 (58.3)	98 (32.3)		
Femur	35 (12.5)	4 (16.7)	39 (12.9)	0.730 [0.211, 2.117]	0.531
Humerus	35 (12.5)	1 (4.2)	36 (11.9)	0.243 [0.026, 1.048]	0.059
Radius/Ulna	125 (44.8)	5 (20.8)	130 (42.9)	0.250 [0.083, 0.660]	0.005
Segment of Long Bone					
Diaphyseal *	197 (70.6)	18 (75.0)	215 (71.0)		
Metaphyseal/Epiphyseal	82 (29.4)	6 (25.0)	88 (29.0)	0.841 [0.308, 2.038]	0.712
Gustilo-Anderson					0.017 **
I *	112 (40.1)	4 (16.7)	116 (38.3)		
II	91 (32.6)	7 (29.2)	98 (32.3)	2.048 [0.632, 7.434]	0.232
IIIA-C	76 (27.2)	13 (54.2)	89 (29.4)	4.411 [1.559, 14.984]	0.004
Vascular Compromise/Injury					
No *	257 (92.1)	20 (83.3)	277 (91.4)		
Yes	22 (7.9)	4 (16.7)	26 (8.6)	2.512 [0.738, 7.092]	0.130
Nerve Injury					
No *	242 (86.7)	23 (95.8)	265 (87.5)		
Yes	37 (13.3)	1 (4.2)	38 (12.5)	0.413 [0.045, 1.674]	0.246
Compartment Syndrome Requiring					
Fasciotomy					
No *	264 (94.6)	21 (87.5)	285 (94.1)		
Yes	15 (5.4)	3 (12.5)	18 (5.9)	2.779 [0.684, 8.776]	0.139

* reference group, ** denotes statistical significance.

**Table 4:** Open Fracture Management Characteristics.

Characteristic Mean (SD), Median (IQR), or *n* (%)	No Infection (*n* = 279)	Deep Infection (*n* = 24)	TOTAL (*n* = 303)	OR [95%OR]	*p*-Value
Time to Hospital (min)	61.8 (±41.2)	89.9 (±64.8)	64.0 (±44.0)	1.009 [1.003, 1.016]	0.008 **
Transferred From OSF					
No *	192 (68.8)	14 (58.3)	206 (68.0)		
Yes	87 (31.2)	10 (41.7)	97 (32.0)	1.594 [0.676, 3.644]	0.280
Time to Antibiotics (min)	161.5 (±157.4)	159.8 (±83.9)	161.4 (±152.8)	1.000 [0.997, 1.002]	0.689
<3 h *	206 (73.8)	18 (75.0)	224 (73.9)		
≥3 h	73 (26.2)	6 (25.0)	79 (26.1)	0.987 [0.361, 2.399]	0.978
Number of Antibiotic Classes Administered	1 (1–2)	2 (1–3)	1 (1–2)	1.271 [0.884, 1.761]	0.185
Time to Operative Debridement (h)	14.5 (±8.5)	18.8 (±27.3)	14.8 (±11.1)	1.021 [0.996, 1.047]	0.093
<6 h *	46 (17.2)	6 (27.3)	52 (17.9)		
≥6 h	222 (82.2)	16 (72.7)	238 (82.1)	0.530 [0.211, 1.486]	0.214
Number of Operative Debridements	1 (1-1)	1 (1-2)	1 (1-1)	1.429 [1.060, 1.893]	0.022 **
Time to Definitive Fixation (h)	23.9 (±36.8)	30.9 (±37.9)	24.5 (±36.9)	1.005 [0.995, 1.012]	0.273
Duration of Antibiotics While Admitted (h)	67.1 (±99.2)	100.3 (±136.1)	69.8 (±102.7)	1.002 [0.999, 1.005]	0.120
Length of Hospital Stay (days)	4.0 (±4.7)	4.8 (±5.7)	4.0 (±4.8)	1.038 [0.954, 1.108]	0.351
Discharged with Antibiotics					
No *	220 (80.0)	18 (81.8)	238 (80.1)		
Yes	55 (20.0)	4 (18.2)	59 (19.9)	0.967 [0.292, 2.611]	0.950
Discharge Disposition					
Home *	265 (95.3)	21 (87.5)	286 (94.7)		
Rehabilitation/Other	13 (4.7)	3 (12.5)	16 (5.3)	3.203 [0.780, 10.319]	0.099
Number of Operative Procedures	1 (1-1)	1 (1-3)	1 (1-1)	1.278 [1.034, 1.584]	0.026 **

* reference group, ** denotes statistical significance.

**Table 5:** Multivariable Analysis for Factors Associated with Developing a Deep Infection After Pediatric Open Long-Bone Fractures.

Characteristic	OR [95% CI]	*p*-Value
Long-Bone		
Tibia/Fibula *		
Femur	0.508 [0.131, 1.614]	0.262
Humerus	236 [0.025, 1.064]	0.062
Radius/Ulna	0.380 [0.107, 1.178]	0.096
Gustilo-Anderson		
I *		
II	1.169 [0.302, 4.845]	0.822
IIIA-C	2.366 [0.622, 10.074]	0.210
Time to Hospital	1.010 [1.003, 1.017]	0.009 **
Number of Operative Debridements	1.209 [0.621, 2.408]	0.573
Number of Operative Procedures	1.001 [0.581, 1.557]	0.996

* reference group, ** denotes statistical significance.

## Data Availability

The data are available in the results section of the manuscript.
